# Annual Herbaceous Plants Exhibit Altered Morphological Traits in Response to Altered Precipitation and Drought Patterns in Semiarid Sandy Grassland, Northern China

**DOI:** 10.3389/fpls.2022.756950

**Published:** 2022-06-23

**Authors:** Shan-Shan Sun, Xin-Ping Liu, Xue-Yong Zhao, Eduardo Medina-Roldánd, Yu-Hui He, Peng Lv, Hong-Jiao Hu

**Affiliations:** ^1^Naiman Desertification Research Station, Northwest Institute of Eco-Environment and Resources, Chinese Academy of Sciences, Lanzhou, China; ^2^University of Chinese Academy of Sciences, Beijing, China; ^3^Urat Desert-Grassland Research Station, Northwest Institute of Eco-Environment and Resources, Chinese Academy of Sciences, Lanzhou, China; ^4^Key Laboratory of Stress Physiology and Ecology in Cold and Arid Regions, Lanzhou, China; ^5^Department of Health and Environmental Sciences, Xi’an Jiaotong-Liverpool University, Suzhou, China

**Keywords:** semiarid sandy grassland, altered precipitation patterns, severe drought stress, annual herbaceous species, morphological traits, biomass allocation patterns

## Abstract

The frequency and intensity of extreme precipitation events and severe drought are predicted to increase in semiarid areas due to global climate change. Plant morphological traits can reflect plant responses to a changing environment, such as altered precipitation or drought patterns. In this study, we examined the response of morphological traits of root, stem, leaf and reproduction meristems of annual herbaceous species to altered precipitation and drought patterns in a semiarid sandy grassland. The study involved a control treatment (100% of background precipitation) and the following six altered precipitation treatments: (1) P(+): precipitation increased by 30%, (2) P(++): precipitation increased by 60%, (3) P(-): precipitation decreased by 30%, (4) P(--): precipitation decreased by 60%, (5) drought 1 (D1): 46-day drought from May 1st to June 15th, and (6) drought 2 (D2): 46-day drought from July 1st to August 15th. P(++) significantly increased root length, flower length-to-width ratio, both P(+) and P(++) significantly increased stem length and flower number in the plant growing seasons, while all of them decreased under P(-) and P(--). The annual herbaceous plants marginally increased the number of second-level stem branches and stem diameter in order to better resist the severe drought stress under P(--). P(+) and P(++) increased the root, stem, leaf, and flower dry weight, with the flower dry weight accounting for a larger proportion than the other aboveground parts. Under D2, the plants used the limited water resources more efficiently by increasing the root-to-shoot ratio compared with P(-), P(--) and D1, which reflects biomass allocation to belowground increased. The linear mixed-effects models and redundancy analysis showed that the root-to-shoot ratio and the dry weight of various plant components were significantly affected by morphological traits and altered precipitation magnitude. Our results showed that the herbaceous species have evolved morphological trait responses that allow them to adapt to climate change. Such differences in morphological traits may ultimately affect the growing patterns of annual herbaceous species, enhancing their drought-tolerant capacity in semiarid sandy grassland during the ongoing climate change.

## Introduction

Global climate change is likely to increase the intensity and frequency of extreme climate events such as severe drought and heavy precipitation at global and regional levels (Smith, 2011; Knapp et al., 2015; Bhusal et al., 2020). Regarding drought, water scarcity can limit plant growth and productivity, with a consequent influence on the persistence and distribution of plant species and negative effects on seedling survival (Choat et al., 2012). Soil water availability is crucial for the various plant growth forms (including grasses, forbs, shrubs, and trees) in terrestrial ecosystems. The extreme response thresholds (outside the bounds of what is considered typical or normal variability) of branches and leaves change with different water availability in grasslands (Smith, 2011), and more frequent precipitation can significantly stimulate the growth of grassland species (Albert et al., 2010; Choat et al., 2012). Climate extremes affect plant growth traits in semiarid grassland ecosystems. For instance, severe drought delays plant flowering time (FT) while increased precipitation may anticipate FT (Huxman et al., 2004; Zhou et al., 2019; Bhusal et al., 2020). Plant morphology is a direct reflection of plants’ interactions with the environment (Wang et al., 2015). Studying how altered precipitation treatments influence plant morphological traits can contribute to the understanding of plant evolutionary growth strategies in semiarid sandy grasslands experiencing ongoing climate change, which has practical implications for landscape management.

As sessile organisms, plants have to continuously respond to changes in the external environment, including by responding with developmental changes, which affects seasonal growth. Grasslands exhibit a symmetric response to wet and dry periods, and grassland vegetation is fairly susceptible to water deficits (Xu et al., 2016). In semiarid sandy grassland, herbaceous species competitively resist environmental factors such as severe drought and desertification. The growth rate of annual herbaceous plants is higher, and more vulnerable to altered precipitation, compared to perennial forbs and grasses in dryland communities (Ogle et al., 2004), with the annual herbaceous species prosperously and dominantly growing in sandy grassland; and there are strong positive correlations between herbaceous plant morphological traits and total summer precipitation amount (Xu et al., 2016). Plant morphological trait variation is the result of evolution, and it has important consequences for community structure and ecosystem function (Huxman et al., 2004; Alonso-Blanco et al., 2009; Ordonez et al., 2009).

Flowering plants consist of roots, stems, leaves, and their outgrowths in the terrestrial ecosystem (Kaplan, 2001; Arnold et al., 2019; Chen et al., 2019b). These plants may modulate their adaptation strategy in response to environmental changes (including climate change) by implementing a variety of morphological changes in these components such as root surface areas (RSA) and stem branches that allow them to function properly and take up limited resources more efficiently (Nicotra et al., 2010; Freschet et al., 2018). This is called phenotypic plasticity and reflects changes in plant dry matter allocation patterns to different components and/or changes in architecture. Key traits (specific root length and leaf area) that exhibit phenotypic plasticity responses can be positively or negatively changed or maintained in response to different intensity gradients of various environmental factors (Niinemets, 2001; Hodge et al., 2009; Comas et al., 2013; Eissenstat et al., 2015; Liu et al., 2015). Studying evolutionary strategies regarding the morphological traits of herbaceous plants can help to predict the drought-tolerance capacity of plants in response to future climate change in semiarid sandy grassland.

The relationships between the root-to-shoot ratio (R/S) and morphological traits of belowground root and aboveground stem, leaf and reproduction change with altered precipitation patterns (McConnaughay and Coleman, 1999; Guo et al., 2017). There is negative correlation between the plant growth rate and R/S (Laughlin and Messier, 2015; Poorter et al., 2015; Huo et al., 2017; Henneron et al., 2019). Aboveground and belowground biomass allocation patterns provide pivotal information for linking the aboveground productivity and belowground carbon sequestration (Bai et al., 2008). Plant biomass allocation patterns of root, stem, leaf and reproduction meristems are strongly correlated with differences in corresponding morphological traits of root, stem, leaf and reproduction meristems at the species level, including aboveground morphological traits such as leaf dry matter content (LDMC; leaf dry weight-to-fresh weight ratio) and specific leaf area (Ordonez et al., 2009), and belowground morphological traits such as specific root length (SRL) and root dry matter content (RDMC; root dry weight-to-fresh weight ratio; Mokany et al., 2006).

This study explored the relationships of R/S, root, stem, leaf, flower dry weight with the aboveground and belowground morphological traits under various precipitation treatments. We did this by conducting a field experiment where the amount of precipitation was manipulated. Specifically, we tested three hypotheses regarding annual herbaceous plants in semiarid sandy grassland: (1) morphological traits of roots, stems, leaves, and reproductive meristems increased with water availability, and were suppressed by decreased precipitation and continuous drought; (2) increased precipitation increased the aboveground biomass proportion, while severe drought increased R/S; and (3) R/S, root, stem, leaf, and flower dry weight were strongly correlated with corresponding morphological traits and precipitation magnitude.

## Materials and Methods

### Experimental Design

The study was conducted in a semiarid sandy grassland in the south-central part of the Horqin Sandy Land (42°55′N, 120°42′E; 360 m elevation), Inner Mongolia, northern China. The site has a continental semiarid monsoon climate and is in a moderate temperature zone. The long-term mean annual temperature and mean annual precipitation (MAP) are about 6.4°C and 360 mm, respectively. Eighty percent of the total precipitation falls from June to August, and the monthly mean temperature ranges from −12.9°C in January to 24.4°C in July. The annual mean wind velocity is 3.2–4.1 m s^−1^, prevailing in the southwest to south and northwest. The soil is zonal and belongs to the chestnut type according to the Chinese classification, and it is a Haplic Calcisol according to the Food and Agriculture Organization (Su et al., 2006). The soil is susceptible to wind erosion, creating denuded soil areas that are then re-colonized by plants (Zuo et al., 2009). This region constitutes a patchwork mosaic characterized by different landscape types (Zuo et al., 2012). Plants that grow well in semiarid sandy grassland include the annual herbaceous species *Euphorbia humifusa* Willd. Enum., *Eragrostis pilosa* (L.) Beauv., *Setaria viridis* (L.) Beauv., *Bassia dasyphylla* (Fisch. et. Mey.) O. Kuntzae, Revis. Gen, *Corispermum macrocarpum* Bge., *Tribulus terretris* L., *Salsola collina* Pall., and the perennial herbaceous species *Cleistogenes squarrosa* (Trin.) Keng, *Pennisetum centrasiaticum* Tzvel., *Phragmites communis* Trrin. Fund., and *Artemisia argyi* Levl. et Vant. (Sun et al., 2019).

The study involved a control treatment (100% of background precipitation) and the following six altered precipitation treatments: precipitation increased by 30% (P(+)) and by 60% (P(++)), precipitation decreased by 30% (P(-)) and by 60% (P(--)), 46-day drought in the seedling stage from May 1st to June 15th (D1) and in the reproductive stage from July 1st to August 15th (D2). All precipitation treatments were designed to fluctuate (± 60% from the mean) based on the regional rainfall amount, and the largest precipitation interval was a single period of 46 days. The other factors related to the plots remained constant, apart from the precipitation treatments, the experiment starting from April in 2011. There were 48 2 × 2 m plots, with 6 per treatment, and 12 in the control group, and with a 2 m buffer zone between each other to avoid the mutual interference of neighbored plots. The decreased precipitation treatment (P(-), P(--), D1, D2) was controlled by sunlight-pervious concave polyvinyl chloride (PVC) boards (1 mm thick) at the 15° angle above each plot, can intercept rainfall of 30% in (P(-)) and 60% in (P(--)), and intercept rainfall of 100% from May 1st to June 15th in D1 and from July 1st to August 15th in D2. Increased precipitation, installed with slotted channels, sprinkled the intercepted water of 30% in (P(+)) and 60% in (P(++)) from corresponding P(-) and P(--) plots immediately after the rain, resulting in 30 and 60% increase relative to ambient precipitation. Generally, the community composition and structure (such as height and cover, species richness, and dominance) in semiarid sandy grassland increased with precipitation increase treatments, thus increasing ecosystem function (such as productivity). Long-term drought events will result in the herbaceous community in semiarid sandy grassland to develop into a single structure, consequently causing the decrease of species diversity and productivity, which is not conducive to the stable recovery of herbaceous species in semiarid sandy grassland (Zhang et al., 2014).

### Species Selection

Two 1 × 1 m quadrats were set up catercorner in each plot to carry out a vegetation survey in the growing season of each year from 2016. We estimated the plant cover and height and recorded the number of plant species in each quadrat. Based on the vegetation survey findings, we used five common annual herbaceous sandy grassland species: *S. viridis*, *Bassia dasyphylla*, *Corispermum macrocarpum*. *Tribulus terretris*, and *S. collina* (Supplementary Table S1) to study morphological traits under altered precipitation treatments. These five herbaceous species are common in sandy grassland in northern China. The importance value (IV) of species in each plot was calculated using the following formula: IV = (RC + RA + RH + RB)/4, where RC is the relative cover of the species (species cover/total cover for all species × 100), RA is the relative abundance (species density/total density for all species × 100), RH is the relative height (species height/total height for all species × 100), and RB is the relative biomass (species biomass/total biomass for all species × 100; Zuo et al., 2012). The total important value (IV) of the five herbaceous species accounted for approximately 54% of all species in the plots, and about 97% of all the annual herbaceous species in the plots (Supplementary Table S2), and the five dominant species can definitely represent the annual herbaceous plant (community).

The morphological traits that we assessed are commonly used to describe plant growth (Supplementary Table S3). Specifically, R/S is commonly used to describe the relationship between the plant belowground and aboveground biomass allocation pattern (Mokany et al., 2006), the leaf length-to-width ratio (LLWR) and specific leaf area (SLA) are closely related to the leaves’ ability to capture light (leaf photosynthesis) and leaf growth, and specific root length (SRL) is closely related to the roots’ ability to obtain resources and root growth (Wright et al., 2004; Roumet et al., 2016).

### Soil Water Content Monitoring

**Figure 1:**
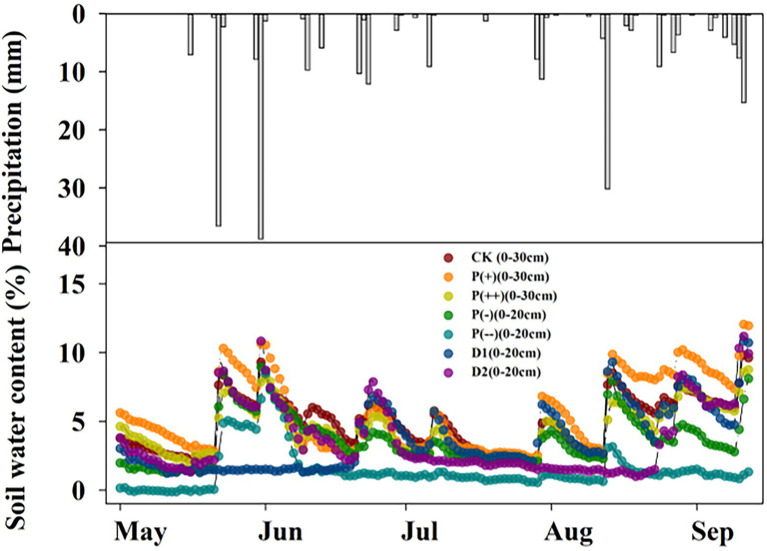
Differences in soil water content at depths of 0–30 cm in precipitation manipulation experiment. CK: control (100% of background precipitation); P(+): 30% increase in precipitation; P(++): 60% increase in precipitation; P(-): 30% decrease in precipitation; P(--): 60% decrease in precipitation; D1: 46-day drought from May 1st to June 15th; D2: 46-day drought from July 1st to August 15th.

ECH_2_O sensors (ECH_2_O-TE; version 1.84; METER Group, Inc., United States), which were set up in experimental plots (each plot having one sensor) in September, 2019, were used to measure the soil water content in each treatment group (measurement frequency range: 5–150 MHz) from September, 2019 to September, 2020. The soil water content at depths of 0–30 cm differed among altered precipitation patterns in 2020, as shown in Figure 1.

### Sampling

We collected at least ten whole plants of each of the five species from each plot at each sampling time (at the time of flowering and immediately before any fruit ripening), respectively on June 15th, July 15th, August 15th, and September 15th in 2020. The sample plants had intact leaves and flowers (fully unfolded), fruits/seeds, stems, and roots. The samples were sandwiched between wet filter paper, placed in self-sealing bags, and then put into a refrigerator in a car (internal temperature < 5°C) to be taken back to be analyzed their morphological traits. The fresh weights of the root, stem, leaf, flower, and fruit plus seed were measured using a Millionth electronic balance (Sartorius; Germany; Zuo et al., 2012). The entire root was spread and scanned using HP ScanJet 2400C (HP, Palo Alto, CA, United States) at 600 dpi and the data were stored as BMP files. We used WinRHIZO (a measuring system to assess root morphology, with a unique overlap correction method) to accurately compute and analyze the total root length (RL) distribution, with the following protocol to ensure high sensitivity: 24-h staining period, root density < 0.5 mm root/mm^2^ surface, resolution of 400 dpi, and automatic threshold (Bouma et al., 2000). Root, stem, leaf, flower, and fruit/seed dry weight were assessed after drying at 60°C for 48 h (Zuo et al., 2012).

### Statistical Analysis

Repeated-measures ANOVAs were performed to test the effects of altered precipitation regimes on morphological traits of annual herbaceous plants in semiarid sandy grassland. One-way ANOVAs with least-significant-difference (LSD) test were used to determine the significance of differences among the precipitation treatments. To assess the changes in relationships between root-to-shoot ratio and plant morphological traits under altered precipitation and drought patterns, we ran linear mixed-effects models (MEMs) using the R package lme4. In the MEMs, the response variable is root-to-shoot ratio and the fixed effect was plant morphological traits (including number of first-level root branches, specific root length, stem length, number of first- and second-level stem branches, leaf diameter, leaf number, flower diameter, flower number) and random effects were altered precipitation and drought patterns and month (Supplementary Table S5). A redundancy analysis (RDA; running in Canoco5) was also used to evaluate the relationships of R/S, root, stem, leaf, and flower dry weight with the morphological traits and the precipitation magnitude (control, P(+), P(++), P(-), and P(--)), as well as the contributive rates of R/S, root, stem, leaf, and flower dry weight explained by morphological traits. All the study data were performed in SPSS 25.0 software.

## Results

### Differences in Morphological Traits Among Precipitation Treatments

**Figure 2:**
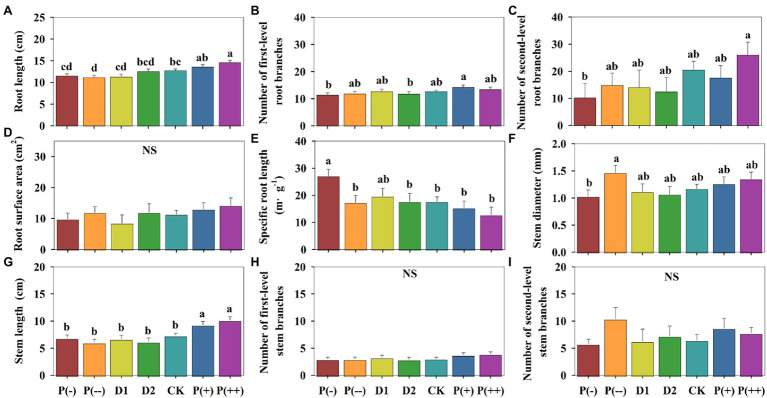
Differences in **(A)** Root length. **(B)** Number of first-level root branches. **(C)** Number of second-level root branches. **(D)** Root surface area. **(E)** Specific root length. **(F)** Stem diameter. **(G)** Stem length. **(H)** Number of first-level stem branches. **(I)** Number of second-level stem branches of annual herbaceous plants in semiarid sandy grassland under altered precipitation and drought patterns. Different lowercase letters indicate significant differences under different precipitation treatments; NS: not significant. CK: control (100% of background precipitation); P(+): 30% increase in precipitation; P(++): 60% increase in precipitation; P(-): 30% decrease in precipitation; P(--): 60% decrease in precipitation; D1: 46-day drought from May 1st to June 15th; D2: 46-day drought from July 1st to August 15th.

**Figure 3:**
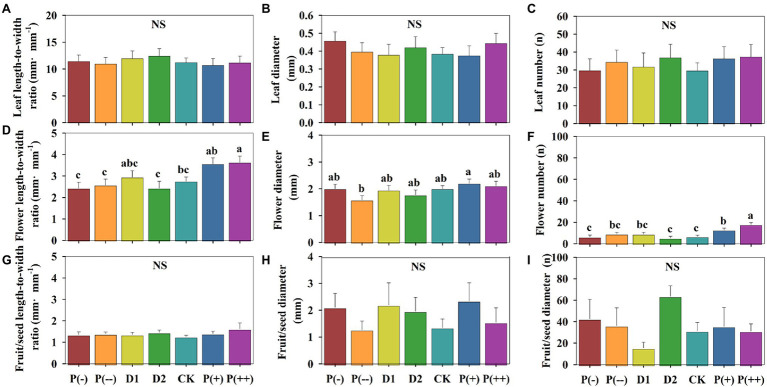
Differences in **(A)** Leaf length-to-width ratio. **(B)** Leaf diameter. **(C)** Leaf number. **(D)** Flower length-to-width ratio. **(E)** Flower diameter. **(F)** Flower number. **(G)** Fruit/seed length-to-width ratio. **(H)** Fruit/seed diameter. **(I)** Fruit/seed number of annual herbaceous plants in semiarid sandy grassland under altered precipitation and drought patterns. Different lowercase letters indicate significant differences under different precipitation treatments; NS: not significant. CK: control (100% of background precipitation); P(+): 30% increase in precipitation; P(++): 60% increase in precipitation; P(-): 30% decrease in precipitation; P(--): 60% decrease in precipitation; D1: 46-day drought from May 1st to June 15th; D2: 46-day drought from July 1st to August 15th.

P(+) and P(++) generally increased the root morphological traits of the five annual herbaceous species in plant growing seasons compared to the natural precipitation treatment, whereas P(-), P(--), D1, and D2 slightly decreased them compared to the natural precipitation treatment (Figure 2). Specifically, P(+) and P(++) increased the root length, number of first-level root branches, root diameter and root surface area in comparison to control; specifically, root length and root diameter significantly increased under P(++; *p* < 0.05, Figure 2 and Supplementary Figure S2). P(-), P(--), D1, and D2 decreased root length, number of first-level and second-level root branches, and root diameter compared with natural treatments; specifically, P(--) significantly decreased root length (*p* < 0.05, Figure 2); P(-) significantly increased the specific root length (*p* < 0.05, Figure 2). The stem morphological traits including stem length, number of first-level and second-level stem branches, and stem diameter (Figure 2), as well as the leaf number (Figure 3), increased under P(+) and P(++) compared with that under CK. P(--) increased number of second-level stem branches and stem diameter, which helped the plants to better adapt to the drought.

There were likewise various effects of the altered precipitation treatments on the morphological traits of plant reproductive meristems. The flower length-to-width ratio (*p* < 0.05 only under P(++)), fruit/seed length-to-width, flower diameter, fruit/seed diameter, flower number (*p* < 0.05), and fruit/seed number increased under P(+) and P(++) compared with that under control (Figure 3). D1 not significantly limited fruit/seed length-to-width and fruit/seed number; D2, however, increased the reproductive (flower and fruit/seed) morphological traits in the peak of the growing season compared with CK, which would impact plant propagation and seed dispersal.

### Differences in Dry Weight Among Precipitation Treatments

**Figure 4:**
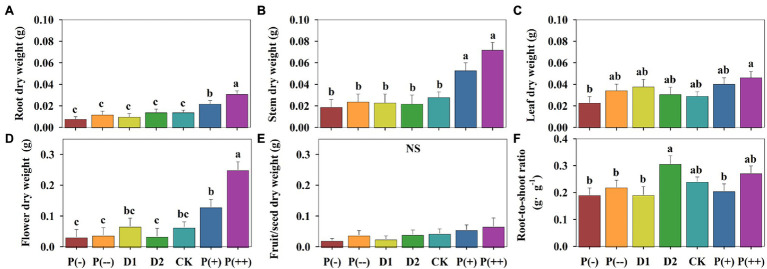
Differences in **(A)** Root dry weight. **(B)** Stem dry weight. **(C)** Leaf dry weight. **(D)** Flower dry weight. **(E)** Fruit/seed dry weight. **(F)** Root-to-shoot ratio of annual herbaceous plants in semiarid sandy grassland under altered precipitation and drought patterns. Different lowercase letters indicate significant differences under different precipitation treatments; NS: not significant. CK: control (100% of background precipitation); P(+): 30% increase in precipitation; P(++): 60% increase in precipitation; P(-): 30% decrease in precipitation; P(--): 60% decrease in precipitation; D1: 46-day drought from May 1st to June 15th; D2: 46-day drought from July 1st to August 15th.

In response to precipitation changes, the annual herbaceous plants allocated different proportions of dry matter (measured in mg/g) to the aboveground stem, leaf, flower, and fruit/seed components and the belowground root component (Figure 4). The root (*p* < 0.05), stem (*p* < 0.05), leaf, and flower (*p* < 0.05 only under P(++)) dry weight increased under increased precipitation treatments in comparison to those under control (Figure 4). In addition, P(+) and P(++) increased the allocation proportion to flower dry weight compared with the control. The root, stem, and fruit/seed dry weight changed little under P(-), P(--), D1, and D2, while leaf dry weight marginally increased under P(--), D1, and D2 compared with that under CK (*p* > 0.05, Figure 4). D2 significantly increased R/S compared with other decreased precipitation (P(-) and P(--)) and drought treatments (D1; *p* < 0.05; Figure 4). In addition, the dry matter contents of root and stem changed slightly with precipitation regime changes (Supplementary Figure S3).

### Relationships of R/S and Dry Weight With Morphological Traits

**Figure 5:**
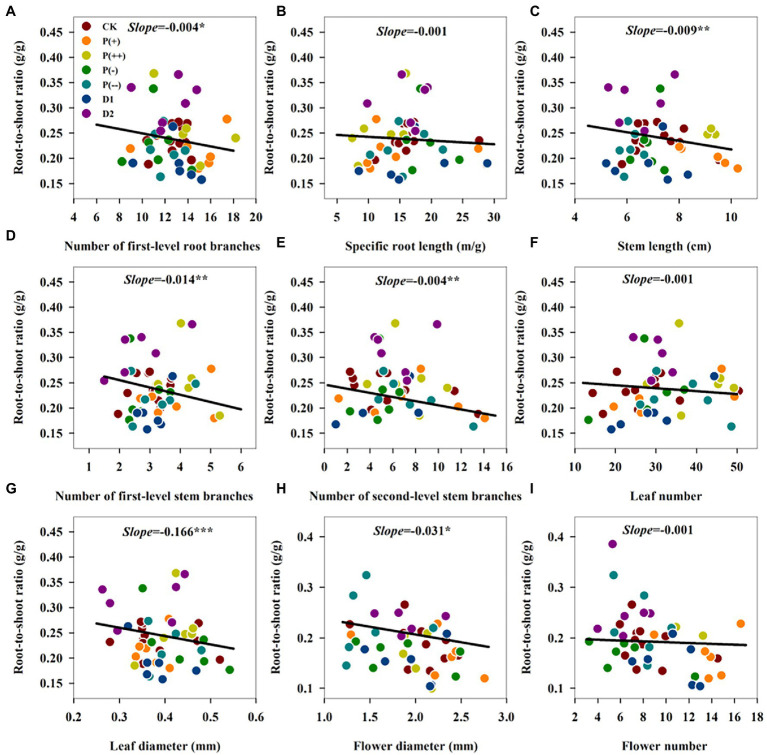
Relationships of **(A)** Number of first-level root branches. **(B)** Specific root length. **(C)** Stem length. **(D)** Number of first-level stem branches. **(E)** Number of second-level stem branches. **(F)** Leaf number. **(G)** Leaf diameter. **(H)** Flower diameter. **(I)** Flower number with root-to-shoot ratio under precipitation regime changes. Each point represents values at a given precipitation intensity. Black line represents the overall relationship from a linear mixed-effects model. The significant level: ^*^p < 0.05; ^**^p < 0.01; ^***^p < 0.001.s

**Figure 6:**
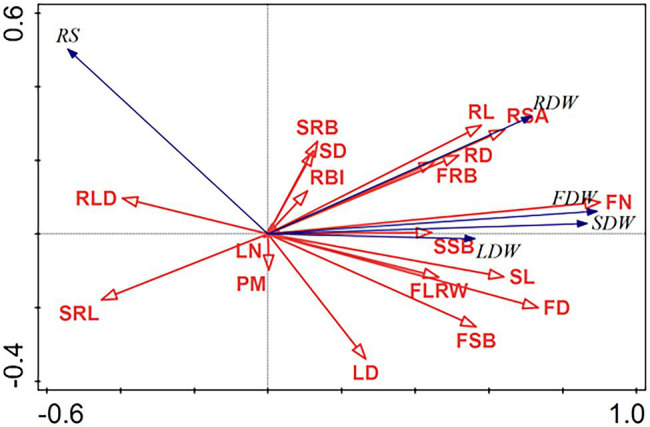
Redundancy analysis (RDA) triplot of the root-to-shoot ratio (R/S), dry weight of root, stem, leaf, and flower, plant morphological traits, and precipitation magnitude (PM). FN: Flower number; FD: Flower diameter; SL: Stem length; SD: Stem diameter; SRL: Specific root length; RSA: Root surface area; RL: Root length; SRB: Number of second-level root branches; LN: Leaf number; FLWR: Flower length-to-width ratio; LD: Leaf diameter; SSB: Number of second-level stem branches; RLD: root length density; RBI: root branch intensity; RD: Root diameter; FRB: Number of first-level root branches; R/S: Root-to-shoot ratio; RDW: Root dry weight; SDW: Stem dry weight; LDW: Leaf dry weight; FDW: Flower dry weight.

The correlation matrix analysis and RDA revealed that the plant biomass allocation patterns were significantly affected by the changes in the morphological traits and altered precipitation magnitude (Table 1; Figure 5). The RDA showed that flower number, flower diameter, stem length, stem diameter, specific root length, and root surface area were the most important factors significantly affecting R/S and plant dry weight of root, stem, leaf and reproduction meristems of herbaceous plant in growing seasons in 2020, explaining 56.8, 6.5, 2.6, 2.0, 2.2, and 1.4%, respectively, of the overall variation in plant biomass allocation (Table 1). R/S was positively correlated with root morphological traits comprising number of second-level root branches, root length density, and root branch intensity, and the stem morphological trait comprising stem diameter. The linear mixed-effects models (MEMs) further showed that the R/S negatively correlated with number of first-level root branches, specific root length, stem length, number of first- and second-level stem branches, leaf number and leaf diameter, flower number and flower diameter (Figure 6). In addition, the RDA showed that the R/S also negatively associated with precipitation magnitude. The root, stem, leaf, and flower dry weight were positively correlated with root surface area, root length, number of first- and second-level root branches, number of first- and second-level stem branches, stem length and diameter, leaf number and diameter, flower number and diameter, while the root and flower dry weight decreased under increased precipitation (Figure 5). The first two RDA axis accounted for 74.44% of the total variation in the plant biomass allocation patterns (Table 1). The first axis accounted for 69.91%, whereas the second only explained 4.53%. The results showed that the biomass allocation patterns of annual herbaceous plants strongly correlated with their morphological traits in semiarid sandy grassland.

**Table 1:** Explained proportions of variance in plant biomass allocation.

	Variable	% Explained	*p* Value	Axis 1	Axis 2
Explained variation (cumulative)				69.91	74.44
Explained fitted variation (cumulative)				90.66	96.54
	Flower number	56.8	0.002		
	Flower diameter	6.5	0.002		
	Stem length	2.6	0.006		
	Stem diameter	2.0	0.01		
	Specific root length	2.2	0.008		
	Root surface area	1.4	0.02		
	Root length	0.7	0.126		
	Number of second-level root branches	0.7	0.154		
	Leaf number	0.5	0.172		
	Precipitation magnitude	0.5	0.218		
	Flower length-to-width ratio	0.5	0.2		
	Leaf diameter	0.5	0.182		
	Number of second-level stem branches	0.4	0.266		
	Root length density	0.3	0.352		
	Root branch intensity	0.3	0.404		
	Root diameter	0.2	0.478		
	Number of first-level stem branches	0.5	0.234		
	Number of first-level root branches	0.5	0.228		

## Discussion

### Annual Herbaceous Plants Exhibited Various Morphological Responses to Altered Precipitation Treatments

Plants are likely to alter their morphological traits to maintain a balance between the water supply and water absorption in semiarid sandy grassland (Niklas and Enquist, 2003; Xu et al., 2007). Precipitation is essential for plant growth, especially in semiarid sandy grassland. Increased precipitation improves water availability and thereby ensures the plant root, stem, and leaf growth and stretching out in the seedling stage (Reich et al., 2003; Messier et al., 2010). Increased precipitation highly increases root morphological traits such as root length and root surface area, thereby enhancing the water use efficiency of herbaceous plants in semiarid sandy grassland (Tomlinson et al., 2012). In our study, P(+) and P(++) increased root length (*p* < 0.05 under P(++)), number of first-level root branches (*p* < 0.05 under P(++)) and root surface area, previous research, however, illustrated that the root size of herbaceous plants decreased with increasing mean annual precipitation (Schenk and Jackson, 2002), suggesting the special capacity of capturing water resource of herbaceous species in semiarid sandy grassland. Root length significantly decreased under P(--) but not P(-), suggesting that highly decreased precipitation limited root growth to ensure aboveground growth. The effects of decreased precipitation (P(-) and P(--)) and continuous drought (D1 and D2) on the morphological traits of herbaceous species differed, as did the effects of light drought (D1 and P(-)) and severe drought (D2 and P(--)) on morphological traits. D1 and P(-) marginally decreased root surface area and number of second-level stem branches (*p* > 0.05), P(-) significantly increased specific root length (*p* < 0.05); whereas D2 and P(--) had the reverse effects on these traits (*p* > 0.05). In semiarid sandy grassland, under light drought (P(-) and D1; with limited water availability), annual herbaceous plant decreased investment in belowground growth and developed the aboveground parts; under heavy drought (P(--) and D2), root surface area increased to exploit the soil water resources and supply aboveground growth (Hodge et al., 2009; Comas et al., 2013; Bhusal et al., 2020).

Plant aboveground morphological traits increased with soil water availability. This is generally in agreement with previous studies on the responses of plant morphological traits to soil water content changes, which demonstrated that leaf and reproductive meristems’ size increased with increased water availability (Niinemets, 2001; Bhusal et al., 2020). Plants have been reported to reduce transpiration by decreasing the leaf area in order to avoid severe water deficiency (Leigh et al., 2017; Wright et al., 2017; Bhusal et al., 2020). However, specific leaf area (showed in Supplementary Figure S2, *p* < 0.05) significantly increased in severe drought, which would improve the ability to capture light energy and increase organic matter synthesis, thereby promoting leaf dry matter accumulation. We observed a larger drought-induced increase in leaf morphological traits (leaf length-to-width ratio and leaf diameter) during the plant reproductive growth stage (D2) than in the seedling stage (D1), while the specific leaf area significantly increased only under D1 (Niinemets, 2001; Wright et al., 2004, 2017; Ordonez et al., 2009). The differences can be explained by water conservation in species growing in arid and semiarid land, as the increasing specific leaf area (leaf area-to-leaf dry weight) with decreased precipitation and severe drought indicates increased drought-tolerant leaf morphology in herbaceous species in semiarid sandy grassland (Niinemets, 2001). Annual herbaceous plants mainly use the shallow surface water (where the soil is moistened by < 5 mm precipitation events) for growth and development. In addition, under decreased precipitation and continuous seasonal drought, the plant life cycle can be accelerated, with more photosynthate allocated to reproductive meristems (Wright et al., 2004, 2017; Ordonez et al., 2009; Leigh et al., 2017).

P(-), P(--), D1, and D2 reduced the herbaceous plant height (referred the belowground height, root length, and aboveground height, stem length) in our study. Some research has found that there was a significant reduction in herbaceous plant belowground height (root length) and root diameter with water deficiency, while other research showed that herbaceous plant belowground height (root length), root diameter, aboveground height (stem length), and stem diameter were not significantly reduced by drought stress (Turcsan et al., 2016; Bhusal et al., 2020). The flower morphological traits of annual herbaceous plants, including flower length-to-width ratio, flower diameter and flower number, increased with water availability, while the fruit/seed morphological traits presented complicated responses to the altered precipitation treatments. Flowering time was positively correlated with plant aboveground height under severe drought due to intraspecific evolution (Zhou et al., 2019); however, the overall flowering phenology of the species assessed was not highly correlated with other morphological traits or the environmental variation (altered precipitation and drought pattern) in our study. D2 marginally increased fruit/seed length-to-width ratio, fruit/seed diameter and number (*p* > 0.05), while D1 decreased fruit/seed number (*p* > 0.05). The results indicate that drought stress of continuous 46 days both in the seedling stage (D1) and in the plant reproductive growth stage (D2) impact plant propagation and seed dispersal (Mccarthy and Enquist, 2007; Turcsan et al., 2016), with D1 decreasing the herbaceous species’ survival.

### Severe Drought Increased R/S

Water availability plays an important role in ecosystem structure and function in semiarid sandy grassland, where limited precipitation suppresses plant growth (Zhou et al., 2019). P(+) and P(++) remarkably increased root (*p* < 0.05), stem (*p* < 0.05), leaf (*p* > 0.05), flower (*p* < 0.05 under P(++)), and fruit/seed dry weight (*p* > 0.05), with flower dry weight accounting for a larger proportion of the aboveground parts than others. Stem, flower, and fruit/seed dry weight decreased under P(-), P(--), D1, and D2 (*p* > 0.05 in these treatments), while leaf dry weight increased in severe drought except that in P(-), with high growth stability. Aboveground net primary production has been shown to be positively correlated with mean annual precipitation, while plants belowground biomass can increase, decrease, or even stay the same with increased MAP (McConnaughay and Coleman, 1999; Mokany et al., 2006; Hodge et al., 2009; Tomlinson et al., 2012; Comas et al., 2013).

Our results showed that only D2 remarkably increased R/S (*p* < 0.05) compared with P(-), P(--), and D1, this indicates that the annual herbaceous species use limited water resources more efficiently by increasing root growth and decreasing aboveground growth to resist severe drought stress in semiarid sandy grassland in July and August. The result was in general agreement with previous research, which demonstrated that the R/S of *Medicago sativa* L. increased with severe drought stress (Zhou et al., 2017). R/S of annual herbaceous plants exhibited different responses to increased precipitation (P(+) and P(++)) and decreased precipitation (P(-) and P(--)), which suggested that the annual herbaceous plant in semiarid sandy grassland had evolved adaptation strategies to these precipitation changes. Some studies have shown that the plant R/S decreased with increasing mean annual precipitation in a grassland ecosystem in northern China (Li et al., 1999; Mccarthy and Enquist, 2007; Tomlinson et al., 2012). However, other studies demonstrated that there was no uniformly changing pattern of R/S with altered precipitation treatments, such as increased R/S and decreased R/S with severe drought stress (Wang et al., 2014; Liu et al., 2015). Herbaceous plants have adopted different response strategies to continuous drought in semiarid sandy grassland: the “defense strategy” allows the plants to allocate more biomass to aboveground components, while the “patience strategy” involves storing more biomass in the plant roots (Chen et al., 2013).

### R/S Was Closely Correlated With Morphological Traits and Precipitation Magnitude

There are positive correlations between dry matter accumulation and plant growth traits in most herbaceous species (Chen et al., 2019a). All the MEMs, correlation analysis and RDA indicated that the R/S allocation patterns closely correlated with morphological traits and precipitation changes, which adds to the findings of previous research (Tomlinson et al., 2012; Poorter et al., 2015). R/S increased with root morphological traits such as number of second-level root branches, while root dry weight was strongly positively correlated with root morphological traits such as root surface area, root diameter, root length, number of first- and second-level root branches and root branch intensity. This high correlation between root dry weight and root morphological traits concurs with previous research (Mccarthy and Enquist, 2007; Hodge et al., 2009; Comas et al., 2013; Eissenstat et al., 2015).

Leaf diameter, as one of the most important plant growth descriptors, determines resource acquisition efficiency and water conservation. Leaf mass per area reflects leaf diameter (Niinemets, 2001), and low leaf mass per area (reflecting low leaf diameter) leads to high water loss because the leaves have thin cuticle layers and shallowly sunken stomata. This is also related to lower foliar photosynthetic rates per unit area (Jagodzinski et al., 2016). In our study, leaf dry matter content changed little under P(-), P(--), and D2, but D1 marginally increased it. Our results illustrated that the impacts on plant root (root surface area), stem (number of second-level stem branches) and reproductive (flower diameter) morphological traits of light drought (D1 and P(-)) differed from those of heavy drought (D2 and P(--)). The differences in morphological traits and the biomass allocation patterns reflect the sandy grassland degradation and recovery process, as there is a compensatory effect between a plant meristem’s dry weight and its-to-area ratio (Li et al., 1999). The results indicated that differences in morphological traits were more important than their biomass allocation pattern in determining species competitive ability. Our study comprehensively investigated the variations in morphological traits and biomass allocation, which will help to predict the competitive outcomes and evolutionary strategies of herbaceous plants during ongoing climate change.

## Conclusion

This study investigated the responses to altered precipitation and drought treatments of morphological traits and dry matter content of annual herbaceous plants in growing seasons. The results showed that increased water availability (P(+) and P(++)) significantly increased certain morphological traits (the root length, stem length, flower length-to-width ratio and number, root, stem and flower dry weight), while number of second-level stem branches and stem diameter could marginally increase to resist decreased precipitation environment (P(--)). The fruit/seed morphological traits changed slightly with altered precipitation and drought treatments, with D1 increasing fruit/seed diameter but decreasing fruit/seed number, and D2 increasing them, which impacts plant propagation and seed dispersal. D2 increased R/S, suggesting that root growth was increased to more efficiently exploit soil water resources under severe drought stress. The MEMs and RDA indicated that morphological traits (especially flower number and diameter, stem length and diameter, specific root length, and root surface area) were key factors impacting the corresponding biomass allocation patterns. The results of this study provide more detailed information for better understanding the morphological evolutionary strategies of annual herbaceous plants in response to altered precipitation and drought treatments. In particular, the findings indicate that annual herbaceous plants can increase morphological traits such as root surface area, number of second-level stem branches and leaf number to better resist severe drought stress during ongoing climate change. Such differences in morphological traits and biomass allocation patterns of herbaceous plants in semiarid sandy grassland may ultimately enhance their drought-tolerant phenotypes.

## Data Availability Statement

The original contributions presented in the study are included in the article/Supplementary Material, further inquiries can be directed to the corresponding author.

## Author Contributions

S-SS designed the research, collected and analyzed the data, and wrote the paper. X-PL and X-YZ assisted with conceived the research idea and revised the paper. EM-R and Y-HH revised the paper. PL assisted with conducted the experiment, collected the data, and conducted the lab analysis. H-JH assisted with collected and analyzed the data. All authors contributed to the article and approved the submitted version.

## Funding

The study was funded by the Science and Technology Poverty Alleviation Project of Chinese Academy of Sciences (KFJ-FP-202104); the Science and Technology Project of Inner Mongolia Autonomous Region (2022YFHH0063), the Strategic Priority Research Program of the Chinese Academy of Sciences (XDA26020104-01).

## Conflict of Interest

The authors declare that the research was conducted in the absence of any commercial or financial relationships that could be construed as a potential conflict of interest.

## Publisher’s Note

All claims expressed in this article are solely those of the authors and do not necessarily represent those of their affiliated organizations, or those of the publisher, the editors and the reviewers. Any product that may be evaluated in this article, or claim that may be made by its manufacturer, is not guaranteed or endorsed by the publisher.

## References

[ref1] Albert C. H. Thuiller W. Yoccoz N. G. Soudant A. Boucher F. Saccone P.. (2010). Intraspecific functional variability: extent, structure and sources of variation. J. Ecol. 98, 604–613. doi: 10.1111/j.1365-2745.2010.01651.x

[ref2] Alonso-Blanco C. Aarts M. G. M. Bentsink L. Keurentjes J. J. B. Reymond M. Vreugdenhil D.. (2009). What has natural variation taught us about plant development, physiology, and adaptation? Plant Cell 21, 1877–1896. doi: 10.1105/tpc.109.068114, PMID: 19574434 PMC2729614

[ref3] Arnold P. A. Kruuk L. E. B. Nicotra A. B. (2019). How to analyse plant phenotypic plasticity in response to a changing climate. New Phytol. 222, 1235–1241. doi: 10.1111/nph.15656, PMID: 30632169

[ref4] Bai Y. Wu J. Xing Q. Pan Q. Huang J. Yang D.. (2008). Primary production and rain use efficiency across a precipitation gradient on the Mongolia plateau. Ecology 89, 2140–2153. doi: 10.1890/07-0992.1, PMID: 18724724

[ref5] Bhusal N. Lee M. Han A. R. Han A. Kim H. S. (2020). Responses to drought stress in *Prunus sargentii* and *Larix kaempferi* seedlings using morphological and physiological parameters. For. Ecol. Manag. 465:118099. doi: 10.1016/j.foreco.2020.118099

[ref6] Bouma T. J. Nielsen K. L. Koutstaal B. (2000). Sample preparation and scanning protocol for computerised analysis of root length and diameter. Plant Soil 218, 185–196. doi: 10.1023/a:1014905104017

[ref7] Chen D. Lan Z. Bai X. Grace J. B. Bai Y. (2013). Evidence that acidification-induced declines in plant diversity and productivity are mediated by changes in below-ground communities and soil properties in a semi-arid steppe. J. Ecol. 101, 1322–1334. doi: 10.1111/1365-2745.12119

[ref8] Chen Y. Shi X. Zhang L. Baskin J. M. Baskin C. C. Liu H.. (2019a). Effects of increased precipitation on the life history of spring- and autumn-germinated plants of the cold desert annual Erodium oxyrhynchum (Geraniaceae). AoB Plants 11:plz004. doi: 10.1093/aobpla/plz004, PMID: 30881621 PMC6410494

[ref9] Chen Y. Zhang L. Shi X. Ban Y. Liu H. Zhang D. (2019b). Life history responses of spring-and autumn-germinated ephemeral plants to increased nitrogen and precipitation in the Gurbantunggut Desert. Sci. Total Environ. 659, 756–763. doi: 10.1016/j.scitotenv.2018.12.368, PMID: 31096405

[ref10] Choat B. Jansen S. Brodribb T. J. Cochard H. Delzon S. Bhaskar R.. (2012). Global convergence in the vulnerability of forests to drought. Nature 491, 752–755. doi: 10.1038/nature11688, PMID: 23172141

[ref11] Comas L. H. Becker S. R. Cruz V. M. V. Byrne P. F. Dierig D. A. (2013). Root traits contributing to plant productivity under drought. Front. Plant Sci. 4:442. doi: 10.3389/fpls.2013.00442, PMID: 24204374 PMC3817922

[ref12] Eissenstat D. M. Kucharski J. M. Zadworny M. Adams T. S. Koide R. T. (2015). Linking root traits to nutrient foraging in arbuscular mycorrhizal trees in a temperate forest. New Phytol. 208, 114–124. doi: 10.1111/nph.13451, PMID: 25970701

[ref13] Freschet G. T. Violle C. Bourget M. Y. Scherer-Lorenzen M. Fort F. (2018). Allocation, morphology, physiology, architecture: the multiple facets of plant above- and below-ground responses to resource stress. New Phytol. 219, 1338–1352. doi: 10.1111/nph.15225, PMID: 29856482

[ref14] Guo Z. Chen D. Alqudah A. M. Roeder M. S. Ganal M. W. Schnurbusch T. (2017). Genome-wide association analyses of 54 traits identified multiple loci for the determination of floret fertility in wheat. New Phytol. 214, 257–270. doi: 10.1111/nph.14342, PMID: 27918076

[ref15] Henneron L. Cros C. Picon-Cochard C. Rahimian V. Fontaine S. (2019). Plant economic strategies of grassland species control soil carbon dynamics through rhizodeposition. J. Ecol. 108, 528–545. doi: 10.1111/1365-2745.13276

[ref16] Hodge A. Berta G. Doussan C. Merchan F. Crespi M. (2009). Plant root growth, architecture and function. Plant Soil 321, 153–187. doi: 10.1007/s11104-009-9929-9

[ref17] Huo C. Luo Y. Cheng W. (2017). Rhizosphere priming effect: A meta-analysis. Soil Biol. Biochem. 111, 78–84. doi: 10.1016/j.soilbio.2017.04.003

[ref18] Huxman T. E. Smith M. D. Fay P. A. Knapp A. K. Shaw M. R. Loik M. E.. (2004). Convergence across biomes to a common rain-use efficiency. Nature 429, 651–654. doi: 10.1038/nature02561, PMID: 15190350

[ref19] Jagodzinski A. M. Dyderski M. K. Rawlik K. Katna B. (2016). Seasonal variability of biomass, total leaf area and specific leaf area of forest understory herbs reflects their life strategies. For. Ecol. Manag. 374, 71–81. doi: 10.1016/j.foreco.2016.04.050

[ref20] Kaplan D. R. (2001). The science of plant morphology: definition, history, and role in modern biology. Am. J. Bot. 88, 1711–1741. doi: 10.2307/3558347, PMID: 21669604

[ref21] Knapp A. K. Hoover D. L. Wilcox K. R. Avolio M. L. Koerner S. E. La Pierre K. J.. (2015). Characterizing differences in precipitation regimes of extreme wet and dry years: implications for climate change experiments. Glob. Chang. Biol. 21, 2624–2633. doi: 10.1111/gcb.12888, PMID: 25652911

[ref22] Laughlin D. C. Messier J. (2015). Fitness of multidimensional phenotypes in dynamic adaptive landscapes. Trends Ecol. Evol. 30, 487–496. doi: 10.1016/j.tree.2015.06.003, PMID: 26122484

[ref23] Leigh A. Sevanto S. Close J. D. Nicotra A. B. (2017). The influence of leaf size and shape on leaf thermal dynamics: does theory hold up under natural conditions? Plant Cell Environ. 40, 237–248. doi: 10.1111/pce.12857, PMID: 28026874

[ref24] Li B. Suzuki J. I. Hara T. (1999). Competitive ability of two Brassica varieties in relation to biomass allocation and morphological plasticity under varying nutrient availability. Ecol. Res. 14, 255–266. doi: 10.1046/j.1440-1703.1999.143298.x

[ref25] Liu B. Li H. Zhu B. Koide R. T. Eissenstat D. M. Guo D. (2015). Complementarity in nutrient foraging strategies of absorptive fine roots and arbuscular mycorrhizal fungi across 14 coexisting subtropical tree species. New Phytol. 208, 125–136. doi: 10.1111/nph.13434, PMID: 25925733

[ref26] Mccarthy M. C. Enquist B. J. (2007). Consistency between an allometric Approach and Optimal Partitioning Theory in Global Patterns of plant Biomass Allocation. Funct. Ecol. 21, 713–720. doi: 10.1111/j.1365-2435.2007.01276.x

[ref27] McConnaughay K. D. M. Coleman J. S. (1999). Biomass allocation in plants: ontogeny or optimality? A test along three resource gradients. Ecology 80, 2581–2593. doi: 10.2307/177242

[ref28] Messier J. McGill B. J. Lechowicz M. J. (2010). How do Traits vary across Ecological scales? A case for trait-based ecology. Ecol. Lett. 13, 838–848. doi: 10.1111/j.1461-0248.2010.01476.x 20482582

[ref29] Mokany K. Raison R. J. Prokushkin A. S. (2006). Critical analysis of root: shoot ratios in terrestrial biomes. Glob. Chang. Biol. 12, 84–96. doi: 10.1111/j.1365-2486.2005.001043.x

[ref30] Nicotra A. B. Atkin O. K. Bonser S. P. Davidson A. M. Finnegan E. J. Mathesius U.. (2010). Plant phenotypic plasticity in a changing climate. Trends Plant Sci. 15, 684–692. doi: 10.1016/j.tplants.2010.09.008, PMID: 20970368

[ref31] Niinemets U. (2001). Global-scale climatic controls of leaf dry mass per area, density, and thickness in trees and shrubs. Ecology 82, 453–469. doi: 10.2307/2679872

[ref32] Niklas K. J. Enquist B. J. (2003). An allometric model for seed plant reproduction. Evol. Ecol. Res. 5, 79–88.

[ref33] Ogle K. Wolpert R. L. Reynolds J. F. (2004). Reconstructing plant root area and water uptake profiles. Ecology 85, 1967–1978. doi: 10.1890/03-0346

[ref34] Ordonez J. C. van Bodegom P. M. Witte J.-P. M. Wright I. J. Reich P. B. Aerts R. (2009). A global study of relationships between leaf traits, climate and soil measures of nutrient fertility. Glob. Ecol. Biogeogr. 18, 137–149. doi: 10.1111/j.1466-8238.2008.00441.x

[ref35] Poorter H. Jagodzinski A. M. Ruiz-Peinado R. Kuyah S. Luo Y. Oleksyn J.. (2015). How does biomass distribution change with size and differ among species? An analysis for 1200 plant species from five continents. New Phytol. 208, 736–749. doi: 10.1111/nph.13571, PMID: 26197869 PMC5034769

[ref36] Reich P. B. Wright I. J. Cavender-Bares J. Craine J. M. Oleksyn J. Westoby M.. (2003). The evolution of plant functional variation: traits, spectra, and strategies. Int. J. Plant Sci. 164, S143–S164. doi: 10.1086/374368

[ref37] Roumet C. Birouste M. Picon-Cochard C. Ghestem M. Osman N. Vrignon-Brenas S.. (2016). Root structure-function relationships in 74 species: evidence of a root economics spectrum related to carbon economy. New Phytol. 210, 815–826. doi: 10.1111/nph.13828, PMID: 26765311

[ref38] Schenk H. J. Jackson R. B. (2002). Rooting depths, lateral root spreads and below-ground/above-ground allometries of plants in water-limited ecosystems. J. Ecol. 90, 480–494. doi: 10.1046/j.1365-2745.2002.00682.x

[ref39] Smith M. D. (2011). An ecological perspective on extreme climatic events: a synthetic definition and framework to guide future research. J. Ecol. 99, 656–663. doi: 10.1111/j.1365-2745.2011.01798.x

[ref40] Su Y. Z. Li Y. L. Zhao H. L. (2006). Soil properties and their spatial pattern in a degraded sandy grassland under post-grazing restoration, inner Mongolia, northern China. Biogeochemistry 79, 297–314. doi: 10.1007/s10533-005-5273-1

[ref41] Sun S.-S. Liu X.-P. He Y.-H. Wei S.-L. Zhang L.-M. Lv P.. (2019). Responses of annual herb plant community characteristics to increased precipitation and reduced wind velocity in semiarid sandy grassland. Ecol. Evol. 9, 10654–10664. doi: 10.1002/ece3.5585, PMID: 31624573 PMC6787865

[ref42] Tomlinson K. W. Sterck F. J. Bongers F. da Silva D. A. Barbosa E. R. M. Ward D.. (2012). Biomass partitioning and root morphology of savanna trees across a water gradient. J. Ecol. 100, 1113–1121. doi: 10.1111/j.1365-2745.2012.01975.x

[ref43] Turcsan A. Steppe K. Sarkoezi E. Erdelyi E. Missoorten M. Mees G.. (2016). Early summer drought stress during the first growing year stimulates extra shoot growth in oak seedlings (*Quercus petraea*). Front. Plant Sci. 7:193. doi: 10.3389/fpls.2016.00193, PMID: 26941760 PMC4763100

[ref44] Wang Q. Chen J. Liu F. Li W. (2014). Morphological changes and resource allocation of *Zizania latifolia* (Griseb.) Stapf in response to different submergence depth and duration. Flora 209, 279–284. doi: 10.1016/j.flora.2014.03.006

[ref45] Wang X. Taub D. R. Jablonski L. M. (2015). Reproductive allocation in plants as affected by elevated carbon dioxide and other environmental changes: a synthesis using meta-analysis and graphical vector analysis. Oecologia 177, 1075–1087. doi: 10.1007/s00442-014-3191-4, PMID: 25537120

[ref46] Wright I. J. Dong N. Maire V. Prentice I. C. Westoby M. Diaz S.. (2017). Global climatic drivers of leaf size. Science 357, 917–921. doi: 10.1126/science.aal4760, PMID: 28860384

[ref47] Wright I. J. Reich P. B. Westoby M. Ackerly D. D. Baruch Z. Bongers F.. (2004). The worldwide leaf economics spectrum. Nature 428, 821–827. doi: 10.1038/nature02403, PMID: 15103368

[ref48] Xu H. Li Y. Xu G. Zou T. (2007). Ecophysiological response and morphological adjustment of two central Asian desert shrubs towards variation in summer precipitation. Plant Cell Environ. 30, 399–409. doi: 10.1111/j.1365-3040.2006.001626.x, PMID: 17324227

[ref49] Xu H.-j. Wang X.-p. Zhang X.-x. (2016). Decreased vegetation growth in response to summer drought in Central Asia from 2000 to 2012. Int. J. Appl. Earth Obs. Geoinf. 52, 390–402. doi: 10.1016/j.jag.2016.07.010

[ref50] Zhang L. Liu X. Zhao X. Zhang T. Yue X. Yun J. (2014). Response of sandy vegetation characteristics to precipitation change in Horqin Sandy land. Acta Ecol. Sin. 34, 2737–2745. doi: 10.5846/stxb201306191738

[ref51] Zhou Z. Li Y. Song J. Ru J. Lei L. Zhong M.. (2019). Growth controls over flowering phenology response to climate change in three temperate steppes along a precipitation gradient. Agric. For. Meteorol. 274, 51–60. doi: 10.1016/j.agrformet.2019.04.011

[ref52] Zhou G. Zhou X. He Y. Shao J. Hu Z. Liu R.. (2017). Grazing intensity significantly affects belowground carbon and nitrogen cycling in grassland ecosystems: a meta-analysis. Glob. Chang. Biol. 23, 1167–1179. doi: 10.1111/gcb.13431, PMID: 27416555

[ref53] Zuo X. A. Knops J. M. H. Zhao X. Y. Zhao H. L. Zhang T. H. Li Y. Q.. (2012). Indirect drivers of plant diversity-productivity relationship in semiarid sandy grasslands. Biogeosciences 9, 1277–1289. doi: 10.5194/bg-9-1277-2012

[ref54] Zuo X. Zhao X. Zhao H. Zhang T. Guo Y. Li Y.. (2009). Spatial heterogeneity of soil properties and vegetation-soil relationships following vegetation restoration of mobile dunes in Horqin Sandy land, northern China. Plant Soil 318, 153–167. doi: 10.1007/s11104-008-9826-7

