# Supplementary material for: Annual Herbaceous Plants Exhibit Altered Morphological Traits in Response to Altered Precipitation and Drought Patterns in Semiarid Sandy Grassland, Northern China
Source: Front Plant Sci. 2022 Jun 23;13:756950. doi: 10.3389/fpls.2022.756950 (PMC9260268; doi:10.3389/fpls.2022.756950)
Supplement: Supplementary file 1 [file Data_Sheet_1.docx]

**Supplementary Material**

***for***

**Annual herbaceous plants exhibit altered morphological traits in response to altered precipitation and drought patterns in semiarid sandy grassland, northern China**

Shan-Shan Sun^1,2,3,4^, Xin-Ping Liu^1*^, Xue-Yong Zhao^1, 3^, Medina-Roldán Eduardo^5^, Yu-Hui He^1^, Peng Lv^1,2^^,3,4^, Hong-Jiao Hu^1, 2^

**This file includes:**

**Figures S1–S3;**

**Tables S1–S6.**


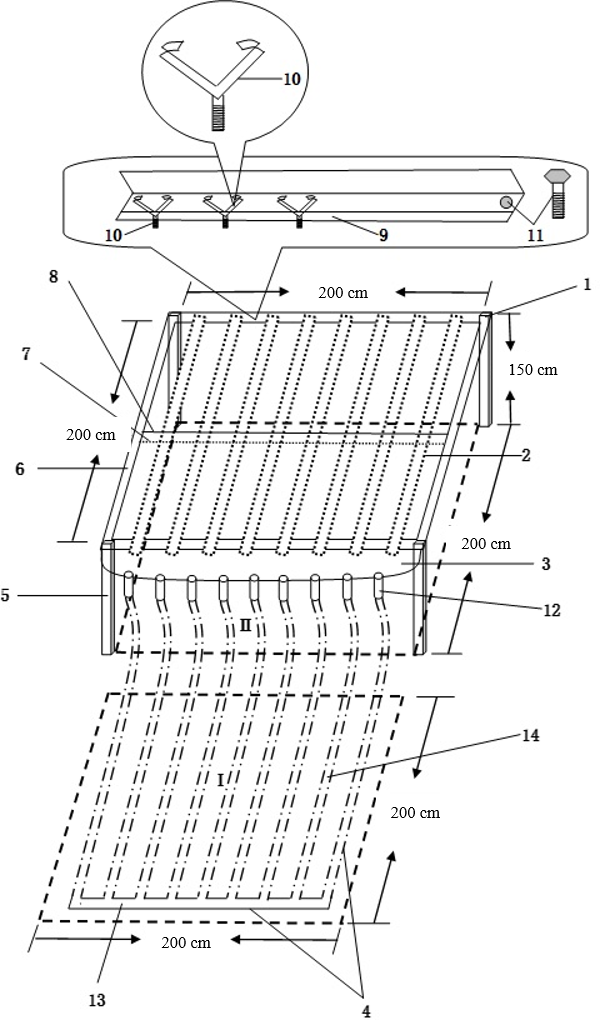


**Figure S1**: The precipitation increase and decrease apparatus. Notes: 1 supporting frame; 2 precipitation interception tough; 3 precipitation collected container; 4 precipitation increase equipment; 5 supporting leg; 6 bracket; 7 stay bar; 8 pressure level; 9 fixed trough; 10 fixed clamp; 11screw; 12 screwed conduit; 13 communicating pipe; 14 precipitation increase pipe.


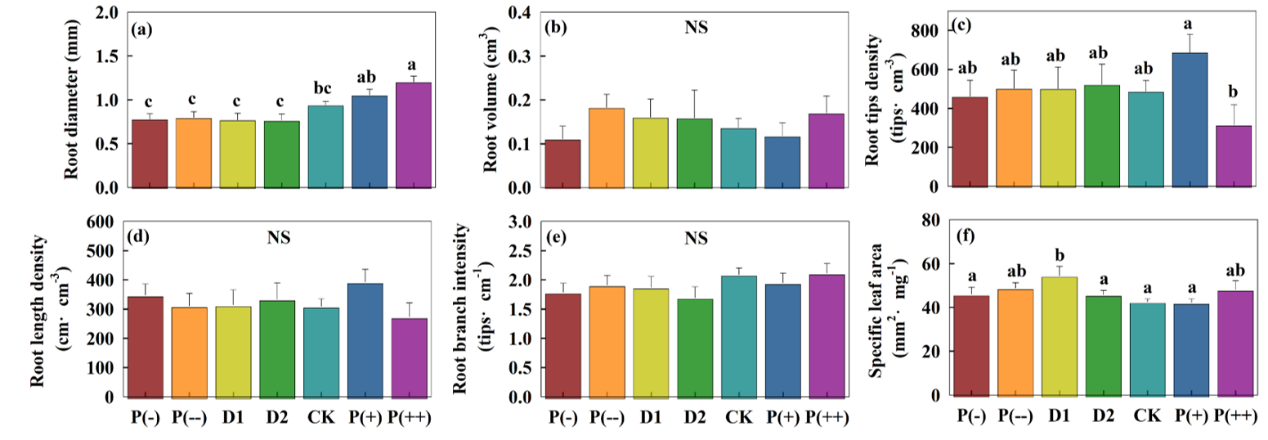


**Figure S2**:| Differences in plant morphological traits of annual herbaceous plants in semiarid sandy grassland under altered precipitation and drought patterns.


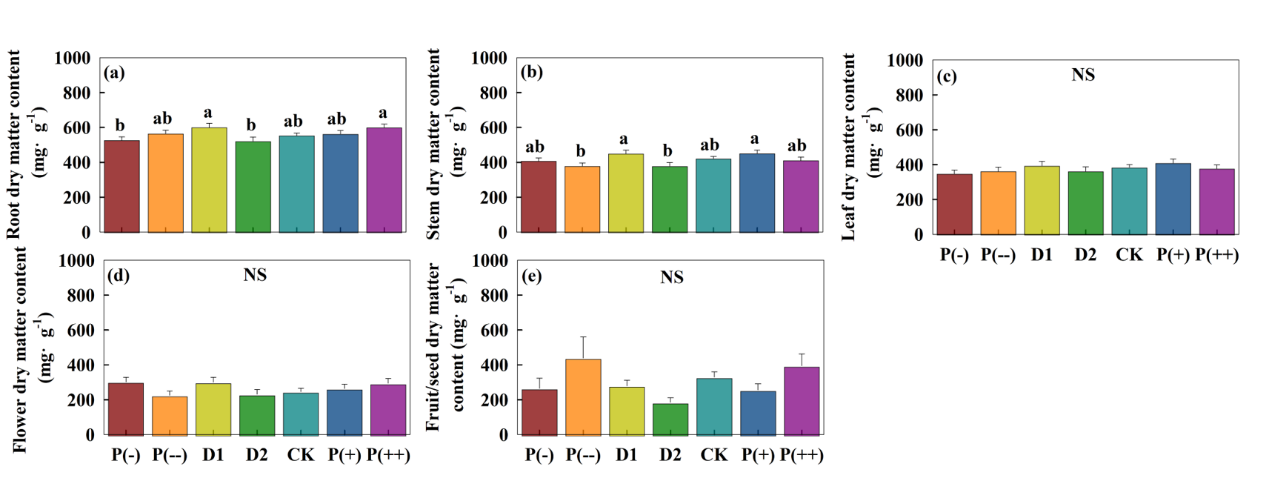


**Figure S3**:| Differences in plant dry matter content of annual herbaceous plants in semiarid sandy grassland under altered precipitation and drought patterns.

**TABLE S1**

| **TABLE S1 \|** The growing characteristics of plant community in rainfall manipulation experiment plots. | | | | | | | | | | | | | | |
| --- | --- | --- | --- | --- | --- | --- | --- | --- | --- | --- | --- | --- | --- | --- |
| **P** | **Species richness** | **May** | | | **June** | | | **July** | | | **August** | | | |
|  |  | **Cover (%)** | **Density (n/m^2^)** | **Height (cm)** | **Cover (%)** | **Density (n/m^2^)** | **Height**  **(cm)** | **Cover (%)** | **Density**  **(n/m^2^)** | **Height**  **(cm)** | **Cover (%)** | **Density**  **(n/m^2^)** | **Height**  **(cm)** | **biomass (g/ m^2^)** |
| CK | 10 | 2.16 | 34.83 | 2.56 | 8.71 | 370.40 | 5.25 | 22.88 | 218.48 | 9.34 | 39.49 | 71.09 | 10.90 | 69.22 |
| P(+) | 11 | 2.91 | 62.92 | 2.80 | 10.09 | 490.67 | 5.51 | 18.99 | 176.40 | 8.66 | 63.38 | 88.25 | 13.39 | 216.07 |
| P(++) | 11 | 2.51 | 34.33 | 2.47 | 7.90 | 523.09 | 5.03 | 18.37 | 282.16 | 8.77 | 36.42 | 51.33 | 10.28 | 56.46 |
| P(-) | 11 | 3.19 | 42.95 | 4.58 | 9.88 | 958.62 | 7.87 | 26.03 | 358.25 | 10.04 | 64.37 | 112.45 | 14.95 | 160.01 |
| P(--) | 10 | 1.70 | 9.75 | 2.92 | 3.44 | 408.32 | 3.09 | 16.14 | 238.08 | 5.47 | 36.19 | 50.83 | 10.11 | 44.12 |
| D1 | 10 | 6.55 | 180.67 | 4.10 | 8.03 | 589.53 | 6.03 | 21.53 | 247.35 | 7.21 | 61.62 | 80.32 | 18.58 | 174.34 |
| D2 | 9 | 2.93 | 53.75 | 2.64 | 7.62 | 683.33 | 4.12 | 19.34 | 357.27 | 5.27 | 32.82 | 50.58 | 7.99 | 56.54 |

Notes: **CK**: control (100% precipitation (P)); **P(+)**: the 30% increase in precipitation; **P(++)**: the 60% increase in precipitation; **P(-)**: the 30% decrease in precipitation; **P(--)**: the 60% decrease in precipitation; **D1**: drought 45 days from May 1^st^ to June 15^th^; **D2**: drought 45 days from July1^st^ to August 15^th^.

**TABLE S2**

| **TABLE S2 \|** The important value (IV) of each species in rainfall manipulation experiment plots. | | | | | | | |
| --- | --- | --- | --- | --- | --- | --- | --- |
| **Species** | **Important Value** | | | | | | |
|  | **CK** | **P(+)** | **P(++)** | **P(-)** | **P(--)** | **D1** | **D2** |
| *A,argyi* |  | 0.39 | 0.09 | 0.26 | 0.09 | 0.36 | 0.04 |
| *Artemisia scoparia Waldst. et Kit.* | 0.24 | 0.17 | 0.20 | 0.07 |  | 0.10 |  |
| *Artemisia sieversiana Ehrh. ex Willd.* |  | 0.01 |  |  |  |  |  |
| *B.dasyphylla* | 0.06 | 0.09 | 0.05 | 0.04 | 0.12 | 0.03 | 0.16 |
| *Chenopodium acuminatum Willd.* | 0.03 | 0.00 |  |  |  |  |  |
| *C.squarrosa* | 0.13 | 0.09 | 0.21 | 0.09 | 0.21 | 0.10 | 0.27 |
| *C.macrocarpum* | 0.22 | 0.20 | 0.15 | 0.14 | 0.20 | 0.18 | 0.16 |
| *Cynanchum theisiodes(Freyn) K. Schum.* | 0.08 | 0.06 | 0.09 | 0.04 | 0.07 | 0.02 | 0.06 |
| *E.humifusa* | 0.02 | 0.03 | 0.03 |  |  |  |  |
| *P.communis* |  | 0.00 |  | 0.14 |  |  |  |
| *S.collina* | 0.06 | 0.06 | 0.04 | 0.05 | 0.08 | 0.05 | 0.09 |
| *S.viridis* | 0.11 | 0.23 | 0.07 | 0.08 | 0.12 | 0.07 | 0.10 |
| *T.terretris* | 0.06 | 0.23 | 0.06 | 0.08 | 0.12 | 0.08 | 0.11 |

Notes: **CK**: control (100% precipitation (P)); **P(+)**: the 30% increase in precipitation; **P(++)**: the 60% increase in precipitation; **P(-)**: the 30% decrease in precipitation; **P(--)**: the 60% decrease in precipitation; **D1**: drought 45 days from May 1^st^ to June 15^th^; **D2**: drought 45 days from July1^st^ to August 15^th^.

The importance value (IV) of species in each plot was calculated using the following formula: IV = (RC+RA+RH+RB) / 4, where RC is the relative cover of the species (species cover / total cover for all species × 100), RA is the relative abundance (species density / total density for all species × 100), RH is the relative height (species height / total height for all species × 100), and RB is the relative biomass (species biomass / total biomass for all species × 100) (Zuo et al., 2012).

**TABLE S3**

| **TABLE S3 \|** Description of the variables studied |  |  |
| --- | --- | --- |
| Abbreviation | Variable | Unit |
| R/S Root-to-shoot ratio g/g  Root morphological traits | | |
| RL | Root length | cm |
| FRB | Number of first-level root branches | N |
| SRB | Number of second-level root branches | N |
| RD | Root diameter | mm |
| RSA | Root surface area | cm^2^ |
| RV | Root volume | cm^3^ |
| SRL | Specific root length | m/g |
| RTID | Root tips density | tips/cm^3^ |
| RDW | Root dry weight | g |
| RDMC | Root dry matter content  (Root dry weight-to-fresh weight ratio) | mg/g |
| Stem morphological traits | | |
| SL | Stem length | cm |
| FSB | Number of first-level stem branches | n |
| SSB | Number of second-level stem branches | n |
| SD | Stem diameter | mm |
| SDW | Stem dry weight | g |
| SDMC | Stem dry matter content  (Stem dry weight-to-fresh weight ratio) | mg/g |
| Leaf morphological traits | | |
| LLWR | Leaf length-to-width ratio | mm/mm |
| LD | Leaf diameter | mm |
| LN | Leaf number | n |
| LDW | Leaf dry weight | g |
| LDMC | Leaf dry matter content  (Leaf dry weight-to-fresh weight ratio) | mg/g |
| Reproductive morphological traits |  |  |
| FLWR | Flower length-to-width ratio | mm/mm |
| FD | Flower diameter | mm |
| FN | Flower number | n |
| FDW | Flower dry weight | g |
| FDMC | Flower dry matter content  (Flower dry weight-to-fresh weight ratio) | mg/g |
| FSLWR | Fruit/seed length-to-width ratio | mm/mm |
| FSD | Fruit/seed diameter | mm |
| FSN | Fruit/seed number | n |
| FSDW | Fruit/seed dry weight | g |
| FSDMC | Fruit/seed dry matter content  (Fruit/seed dry weight-to-fresh weight ratio) | mg/g |

**TABLE S****4**

| **TABLE S4 \|** Correlations of R/S and plant dry weights with precipitation and morphological traits | | | | | | |
| --- | --- | --- | --- | --- | --- | --- |
|  | R/S | RDW | SDW | LDW | FDW | FSDW |
| Precipitation magnitude | 0.166** | -0.090* | -0.124** | -0.082* | -0.027 | 0.166 |
| RL | -0.066 | 0.501** | 0.462** | 0.340** | 0.477** | 0.193 |
| FRB | -0.251** | 0.518** | 0.497** | 0.405** | 0.461** | 0.093 |
| SRB | -0.019 | 0.569** | 0.422** | 0.523** | 0.185** | 0.014 |
| RD | -0.044 | 0.749** | 0.676** | 0.596** | 0.441** | 0.206* |
| RSA | 0.014 | 0.274** | 0.233** | 0.159** | 0.223** | -0.019 |
| RD | 0.047 | 0.120** | 0.102** | 0.073 | 0.097 | 0.212* |
| RV | 0.050 | 0.090* | 0.072 | 0.044 | 0.07 | 0.002 |
| SRL | -0.059 | -0.345** | -0.280** | -0.279** | -0.260** | -0.188 |
| RLD | -0.014 | -0.249** | -0.185** | -0.175** | -0.151** | -0.178 |
| RTD | -0.144** | 0.066 | 0.129** | 0.064 | -0.034 | -0.145 |
| RBI | -0.158** | 0.438** | 0.385** | 0.343** | 0.184** | -0.052 |
| SL | -0.156** | 0.708** | 0.717** | 0.494** | 0.501** | .258** |
| FSB | -0.158** | 0.459** | 0.533** | 0.430** | 0.367** | 0.051 |
| SSB | -0.241** | 0.278** | 0.410** | 0.509** | 0.138 | 0.543** |
| SD | -0.016 | 0.267** | 0.250** | 0.224** | 0.220** | 0.230* |
| LN | -0.119** | 0.458** | 0.497** | 0.667** | 0.047 | 0.373** |
| LLWR | 0.024 | -0.008 | -0.056 | 0.008 | -0.071 | -0.058 |
| LD | -0.230** | 0.003 | 0.034 | 0.219** | 0.102* | -0.001 |
| FLWR | 0.000 | 0.382** | 0.314** | 0.317** | 0.201** | 0.155 |
| FD | -0.377** | 0.105* | 0.148** | -0.017 | 0.458** | -0.026 |
| FN | -0.304** | 0.677** | 0.744** | 0.591** | 0.718** | 0.138 |
| FSLWR | 0.054 | 0.004 | -0.017 | 0.049 | 0.116 | -0.051 |
| FSD | 0.065 | -0.130 | -0.179 | -0.159 | -0.212 | 0.326** |
| FSN | 0.029 | 0.233* | 0.237* | 0.399** | 0.001 | 0.309** |

Notes: *, *P* < 0.05; **, *P* < 0.01; ***, *P* < 0.001.

**TABLE S5**

| **TABLE S5 \|** Mixed-effects for linear mixed-effects models (LMMs) from Fig. 5, with “month” and “altered precipitation and drought patterns” as random effects. | | | | | | | | | |
| --- | --- | --- | --- | --- | --- | --- | --- | --- | --- |
| NO. | Dependent variables | Independent variables | Estimate | Std. Error | *t-values* | *P-values* | Lower Bound | Upper Bound | Models |
| 1 | Root-to-shoot ratio | Root length | 0.001 | 0.003 | *0.365* | *0.7157* | *-0.005* | *0.008* |  |
|  |  | (Intercept) | 0.222 | 0.045 | *4.971* | *0.0000* | *0.134* | *0.310* |  |
| 2 | Root-to-shoot ratio | Number of first-level root branches | -0.004 | 0.002 | *-2.440* | ***0.0158*** | *-0.008* | *-0.001* | Fig.5a |
|  |  | (Intercept) | 0.293 | 0.026 | *11.239* | *0.0000* | *0.241* | *0.344* |  |
| 3 | Root-to-shoot ratio | Number of second-level root branches | -0.001 | 0.001 | *-0.949* | *0.3446* | *-0.002* | *0.001* |  |
|  |  | (Intercept) | 0.222 | 0.016 | *13.586* | *0.0000* | *0.189* | *0.255* |  |
| 4 | Root-to-shoot ratio | Root diameter | -0.021 | 0.026 | *-0.799* | *0.4254* | *-0.072* | *0.031* |  |
|  |  | (Intercept) | 0.257 | 0.028 | *9.172* | *0.0000* | *0.201* | *0.312* |  |
| 5 | Root-to-shoot ratio | Root surface area | 0.000 | 0.001 | *0.323* | *0.7468* | *-0.001* | *0.002* |  |
|  |  | (Intercept) | 0.237 | 0.017 | *13.947* | *0.0000* | *0.203* | *0.271* |  |
| 6 | Root-to-shoot ratio | Root volume | 0.054 | 0.048 | *1.115* | *0.2662* | *-0.041* | *0.149* |  |
|  |  | (Intercept) | 0.234 | 0.016 | *15.059* | *0.0000* | *0.202* | *0.265* |  |
| 7 | Root-to-shoot ratio | Specific root length | -0.001 | 0.001 | *-1.277* | *0.2031* | *-0.002* | *0.000* | Fig.5b |
|  |  | (Intercept) | 0.250 | 0.018 | *13.699* | *0.0000* | *0.213* | *0.287* |  |
| 8 | Root-to-shoot ratio | Root tips density | 0.000 | 0.000 | *1.347* | *0.1796* | *0.000* | *0.000* |  |
|  |  | (Intercept) | 0.221 | 0.019 | *11.367* | *0.0000* | *0.183* | *0.260* |  |
| 9 | Root-to-shoot ratio | Root length density | 0.000 | 0.000 | *-0.141* | *0.8883* | *0.000* | *0.000* |  |
|  |  | (Intercept) | 0.241 | 0.018 | *13.597* | *0.0000* | *0.206* | *0.277* |  |
| 10 | Root-to-shoot ratio | Root branch intensity | -0.022 | 0.007 | *-3.085* | ***0.0025*** | *-0.036* | *-0.008* |  |
|  |  | (Intercept) | 0.281 | 0.019 | *14.904* | *0.0000* | *0.243* | *0.319* |  |
| 11 | Root-to-shoot ratio | Stem length | -0.009 | 0.003 | *-3.231* | *0.0016* | *-0.014* | *-0.003* | Fig.5c |
|  |  | (Intercept) | 0.303 | 0.024 | *12.768* | *0.0000* | *0.255* | *0.350* |  |
| 12 | Root-to-shoot ratio | Number of first-level stem branches | -0.014 | 0.005 | *-3.113* | *0.0022* | *-0.024* | *-0.005* | Fig.5d |
|  |  | (Intercept) | 0.284 | 0.020 | *14.544* | *0.0000* | *0.245* | *0.323* |  |
| 13 | Root-to-shoot ratio | Number of second-level stem branches | -0.004 | 0.001 | *-3.475* | *0.0008* | *-0.006* | *-0.002* | Fig.5e |
|  |  | (Intercept) | 0.247 | 0.015 | *16.559* | *0.0000* | *0.216* | *0.277* |  |
| 14 | Root-to-shoot ratio | Stem diameter | -0.018 | 0.017 | *-1.066* | *0.2880* | *-0.052* | *0.015* |  |
|  |  | (Intercept) | 0.260 | 0.026 | *10.164* | *0.0000* | *0.209* | *0.310* |  |
| 15 | Root-to-shoot ratio | Leaf length-to-width ratio | -0.001 | 0.000 | *-1.571* | *0.1179* | *-0.001* | *0.000* |  |
|  |  | (Intercept) | 0.257 | 0.019 | *13.478* | *0.0000* | *0.219* | *0.295* |  |
| 16 | Root-to-shoot ratio | Leaf number | -0.047 | 0.057 | *-0.823* | *0.4114* | *-0.161* | *0.066* | Fig.5f |
|  |  | (Intercept) | 0.256 | 0.027 | *9.426* | *0.0000* | *0.202* | *0.310* |  |
| 17 | Root-to-shoot ratio | Leaf diameter | -0.001 | 0.002 | *-0.364* | *0.7166* | *-0.005* | *0.003* | Fig.5g |
|  |  | (Intercept) | 0.246 | 0.028 | *8.732* | *0.0000* | *0.190* | *0.302* |  |
| 18 | Root-to-shoot ratio | Flower length-to-width ratio | 0.001 | 0.008 | 0.074 | 0.9413 | -0.015 | 0.016 |  |
|  |  | (Intercept) | 0.187 | 0.032 | 5.891 | 0.0000 | 0.123 | 0.252 |  |
| 19 | Root-to-shoot ratio | Flower diameter | -0.031 | 0.012 | -2.639 | 0.0114 | -0.054 | -0.007 | Fig.5h |
|  |  | (Intercept) | 0.268 | 0.032 | 8.274 | 0.0000 | 0.203 | 0.334 |  |
| 20 | Root-to-shoot ratio | Flower number | -0.001 | 0.001 | -0.703 | 0.4864 | -0.003 | 0.002 | Fig.5i |
|  |  | (Intercept) | 0.199 | 0.020 | 9.856 | 0.0000 | 0.156 | 0.243 |  |

**TABLE S6**

| **TABLE S6 \|** The precipitation magnitude (PM) in different experiment treatments during the growing seasons. | | | | |
| --- | --- | --- | --- | --- |
| PM (mm) | June | July | August | September |
| CK | 110.00 | 36.00 | 55.60 | 24.60 |
| P(+) | 143.00 | 46.80 | 72.28 | 31.98 |
| P(++) | 176.00 | 57.60 | 88.96 | 39.36 |
| P(-) | 77.00 | 25.20 | 38.92 | 17.22 |
| P(--) | 44.00 | 14.40 | 22.24 | 9.84 |
| D1 | 0.00 | 36.00 | 55.60 | 24.60 |
| D2 | 110.00 | 26.20 | 0.00 | 24.60 |
